# Decreased H3K27 and H3K4 trimethylation on mortal chromosomes in distributed stem cells

**DOI:** 10.1038/cddis.2014.522

**Published:** 2014-12-04

**Authors:** Y H Huh, J L Sherley

**Affiliations:** 1 The Adult Stem Cell Technology Center, LLC, Boston, MA, USA; 2 Division of Electron Microscopic Research, Korea Basic Science Institute, 169-148 Gwahak-ro, Yuseong-gu, Daejeon, Korea

## Abstract

The role of immortal DNA strands that co-segregate during mitosis of asymmetrically self-renewing distributed stem cells (DSCs) is unknown. Previously, investigation of immortal DNA strand function and molecular mechanisms responsible for their nonrandom co-segregation was precluded by difficulty in identifying DSCs and immortal DNA strands. Here, we report the use of two technological innovations, selective DSC expansion and establishment of H2A.Z chromosomal asymmetry as a specific marker of ‘immortal chromosomes,' to investigate molecular properties of immortal chromosomes and opposing ‘mortal chromosomes' in cultured mouse hair follicle DSCs. Although detection of the respective suppressive and activating H3K27me3 and H3K4me3 epigenetic marks on immortal chromosomes was similar to randomly segregated chromosomes, detection of both was lower on mortal chromosomes destined for lineage-committed sister cells. This global epigenomic feature of nonrandom co-segregation may reveal a mechanism that maintains an epigenome-wide ‘poised' transcription state, which preserves DSC identity, while simultaneously activating sister chromosomes for differentiation.

Distributed stem cells (DSCs) are responsible for the continuous replenishment of the short-lived differentiating cells of renewing tissues.^1, 2, 3, 4, 5, 6, 7, 8, 9^ DSCs accomplish this role by their unique asymmetric self-renewal, a cell division program that preserves stem cell phenotype with simultaneous production of cells committed to tissue-specific differentiation lineages.^2, 3^ Tightly associated with asymmetric self-renewal, DSCs adopt a nonrandom form of mitotic chromosome segregation defined by continuous co-segregation of the set of chromosomes with the older template DNA strands to asymmetrically dividing DSCs.^7, 10, 11^ These oldest DNA strands in asymmetrically self-renewing DSCs are called ‘immortal strands',^1^ and the chromosomes bearing them are called ‘immortal chromosomes'.^6^ Immortal DNA strand co-segregation has been detected in an increasingly diverse range of tissues and vertebrate species.^7, 12, 13, 14, 15, 16, 17, 18, 19, 20, 21, 22, 23, 24^

Nonrandom sister chromatid segregation (that is, co-segregation of immortal chromosomes) was originally proposed as a mechanism that could limit DSC gene mutations that arise from DNA replication errors.^1^ More recently, it has been suggested to have a role in preserving the stemness phenotype of DSCs during asymmetric self-renewal divisions,^25^ and accumulated damage to long-lived immortal DNA strands in DSCs has been proposed as a mechanism of tissue aging.^2^ However, neither the exact cellular function(s) of immortal chromosomes nor the mechanisms responsible for their nonrandom co-segregation are known.

Previously, investigations of the function of nonrandom co-segregation of immortal chromosomes and the responsible molecular mechanisms were largely precluded because of difficulty in obtaining DSCs in the quantity and purity required for meaningful molecular analyses and the lack of direct assays for detection of immortal DNA strands or their immortal chromosomes. Toward enabling molecular investigations of immortal DNA strands, we addressed the DSC scarcity problem by developing a general method for selective *ex vivo* expansion of diverse DSCs. This method is based on suppressing the asymmetric cell kinetics (suppression of asymmetric cell kinetics; ‘SACK') of DSCs.^4, 5, 7^ The DSC fraction of cultures of the SACK-derived, adult mouse hair follicle DSCs used in the presented studies is estimated to be⩾44%. The derivation of these clonal DSC strains has been described previously.^7^ In brief, they were derived from cell cultures outgrown from isolated mouse whisker follicles incubated in medium supplemented with the SACK agent xanthine (Xn). Before culture, whisker follicles were first cleared of all external cellular material and then slit with a small incision to allow follicle cells to divide and escape. Subsequently, cell strains with Xn-dependent growth were clonally derived. Their DSCs properties include purine-dependent asymmetric self-renewal, purine-dependent nonrandom sister chromatid segregation, long-term self-renewal, and production of multiple differentiated cell types of the skin and hair follicles.^7^

For much of their history, immortal DNA strands were mainly detected with retrospective assays based on their labeling properties with DNA base analogs.^7, 12, 13, 14, 15, 17, 18, 19, 20, 21, 22, 23, 24^ With one recent exception,^21, 24^ these assays require chromosome denaturants that would confound investigations of molecular properties of immortal chromosomes that might illuminate their cellular function and components of the mechanism of nonrandom co-segregation. Recently, we reported the histone H2A variant H2A.Z as a pattern-specific, prospective biomarker for mouse immortal chromosomes. In both engineered mouse cell lines and mouse hair follicle DSCs undergoing nonrandom co-segregation, by immunofluorescence, H2A.Z is detected primarily on immortal chromosomes. The H2A.Z chromosomal asymmetry assay has 89% concordance with traditional DNA base analog assays for detection of immortal chromosomes.^6^

The combination of SACK-expanded cell strains highly enriched for DSCs and H2A.Z as a native biomarker for immortal chromosomes is a technical advance that enables a new approach to investigation of the molecular basis of nonrandom chromosome co-segregation in DSCs. This approach is interrogation of DSCs for proteins and other labile cellular factors that are detected specifically on either immortal chromosomes or mortal chromosomes. Such distinctive patterns of detection could indicate specific structural or functional features of the mechanisms responsible for nonrandom co-segregation and associated asymmetric self-renewal by DSCs. Here, we report the first trial of this newly available strategy. We find that two well-described molecular marks of gene regulation, trimethylation of histone H3 lysines 27 and 4, display such an asymmetric distribution of detection between immortal chromosomes and mortal chromosomes in asymmetrically self-renewing mouse hair follicle-derived DSCs.

## Results

Using H2A.Z asymmetry to identify immortal chromosomes in SACK-expanded mouse hair follicle DSCs, we applied specific monoclonal antibodies in indirect *in situ* immunofluorescence (ISIF) analyses to investigate the respective chromosomal content of trimethylation on histone H3 lysine 27 (H3K27me3) and lysine 4 (H3K4me3). These epigenetic modifications were evaluated because of their reported roles in transcriptional suppression and activation, respectively.^8^ To insure the specificity of respective H2A.Z and H3K27me3 or H3K4me3 fluorescence signals, control analyses were performed that omitted each primary antibody individually or both together. In addition, antigen-blocking experiments were performed with respective peptide epitope antigen for H3K27me3 and H3K4me3 antibodies. The specificity of the H2A.Z antibodies was confirmed in this manner in an earlier study.^6^ Pre-incubation of the anti-H3K27me3 antibodies at room temperature for 1 h with 0.1 *μ*g of H3K27me3 peptide epitope antigen ablated anti-H3K27me3-dependent fluorescence. Similarly, pre-incubation of the anti-H3K4me3 antibodies at room temperature for 1 h with 0.1 *μ*g of H3K4me3 peptide epitope antigen ablated anti-H3K4me3-dependent fluorescence (Supplementary Fig. S1,Supplementary Information).

**Figure 1:**
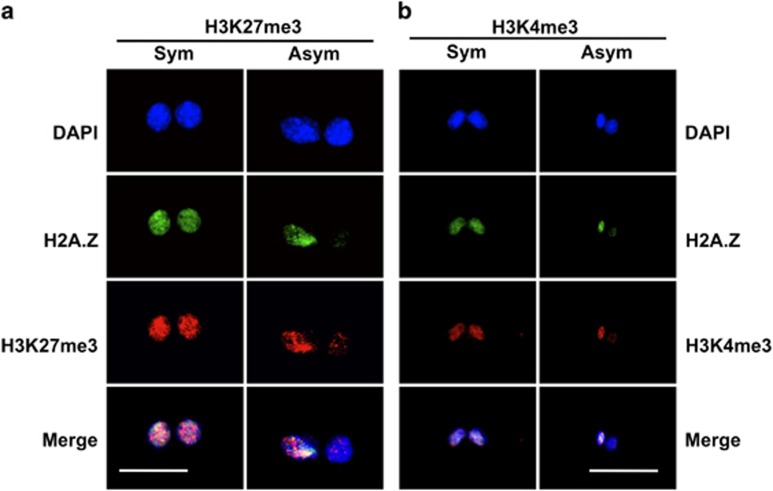
Respective association of chromosomal H3K27me3 and H3K4me3 detection level with H2A.Z-positive immortal chromosomes in murine hair follicle DSCs undergoing nonrandom co-segregation. Shown are examples of CD-arrested binucleated 3C5 cells, grown under conditions that promote nonrandom co-segregation, illustrating either symmetric (Sym) or asymmetric (Asym) detection of chromosomal H2A.Z by ISIF. (**a**) Examples of co-symmetric and co-asymmetric association of H3K27me3 detection level (**b**). Examples of co-symmetric and co-asymmetric association of H3K4me3 detection level. DAPI, nuclear DNA fluorescence. Merge, overlay of above three fluorescence images. Scale bar=25 microns

**Figure 2:**
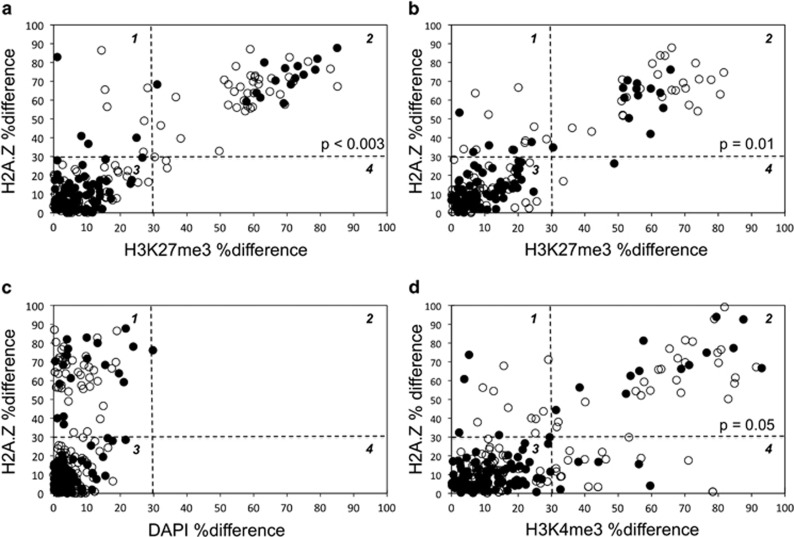
Quantitative analyses of the associated detection of H2A.Z chromosomal asymmetry with respective H3K27me3 and H3K4me3 chromosomal asymmetry during nonrandom co-segregation. Strain 3C5 (**a**, **c**, **d**) and strain 5B8 (**b**) murine hair follicle DSCs were evaluated under conditions that either promoted (Xn-free; open circles) or suppressed (Xn-supplemented; closed circles) nonrandom co-segregation. After CD-arrest, binucleated cells were evaluated by quantitative ISIF with anti-H2A.Z antibodies in combination with respective anti-H3K27me3 antibodies (**a**, **b**) or anti-H3K4me3 antibodies (**d**). The percent difference in mean fluorescence intensity (%difference) for H2A.Z detection between sister nuclei in each examined binucleated cell is plotted against the corresponding %difference for either H3K27me3 detection (**a**, **b**) or H34Kme3 detection (**d**). In **c**, the corresponding distribution of the %difference in DAPI mean fluorescence intensity for the same binucleated cells evaluated in **a** was use to set thresholds for chromosomal detection patterns. Based on this analysis, a %difference ⩽30% was used to define a symmetric chromosomal detection pattern; and a %difference >30% defined an asymmetric chromosomal detection pattern. Quadrant 2 data correspond to binucleated cells exhibiting dual asymmetry. *P*-values indicate the level of statistical confidence, from the two-tailed Fisher's exact test, that the observed reduction in binucleated cells with dual asymmetry under conditions that suppress nonrandom co-segregation is not a chance occurrence

The previously described cytochalasin D (CD)-arrest assay was used for these studies, because of its advantage of exact sister–sister comparisons of nuclear proteins.^6, 7, 10, 11, 26^ Like H2A.Z, both epigenetic marks also showed examples of CD-arrested binucleated cells with significant chromosomal asymmetry (Figure 1, Asym). On the basis of the control distribution of %differences between the 4'-6-diamido-2-phenylindole (DAPI) mean fluorescence intensity (MFI) of sister nuclei in CD-arrested cells (See Figure 2c), for quantitative analysis, chromosomal asymmetry was defined as a %difference in MFI >30%. Reciprocally, a symmetric chromosomal detection pattern was defined as ⩽30% difference.

CD-arrested cells that displayed H3K27me3 asymmetry or H3K4me3 asymmetry also had a high rate of associated H2A.Z chromosomal asymmetry (Figure 1, Asym; Figure 2, compare quadrant 2 for a, b, and d with c). The frequencies of both types of cells with dual chromosomal asymmetry were significantly reduced by Xn supplementation. These properties are indicative of a dependency on nonrandom co-segregation, which is highly associated with H2A.Z asymmetry^6^ and suppressed by the guanine ribonucleotide precursor Xn.^7, 11^ Under Xn-free conditions, which foster nonrandom co-segregation, strains 3C5 and 5B8 cells had respective 32% (Figure 2a, quadrant 2, open circles) and 30% (Figure 2b, quadrant 2, open circles) frequencies of CD-arrested cells displaying both H3K27me3 asymmetry and H2A.Z asymmetry. Xn-supplementation reduced these frequencies to 15% (Figure 2a, quadrant 2, closed circles) and 13% (Figure 2b, quadrant 2, closed circles), respectively.

To a lesser but significant extent, examination of strain 3C5 revealed that it also produced cells with associated H3K4me3 and H2A.Z asymmetry (Figure 2d, quadrant 2). Cells with this feature decreased from 21% (Figure 2d, quadrant 2, open circles) to 11% (Figure 2d, quadrant 2, closed circles) when cultures were cultured in Xn-supplemented medium. We confirmed that the observed dual marker asymmetry was not due to interference between co-evaluating antibodies. In the case of H2A.Z and H3K27me3 dual asymmetry, we confirmed that the frequency of 3C5 DSCs with H3K27me3 asymmetry was similar when the anti-H3K27me3 antibodies were used alone (25%) or in combination with anti-H2A.Z antibodies, whether they were bound first (as routinely; See Materials and Methods; 33%) or second in the procedure (26%).

Under Xn-free conditions, which maximize nonrandom co-segregation, 97% of cells showing H3K27me3 asymmetry also showed H2A.Z asymmetry (Figures 2a and b, compare quadrant 2 with quadrant 4); and 62% of cells exhibiting H3K4me3 asymmetry also showed H2A.Z asymmetry (Figure 2d, compare quadrant 2 with quadrant 4). Direct inspection of ISIF images (as in Figure 1, Asym) demonstrated that essentially all binucleated cells with both H2A.Z asymmetry and respective H3 trimethylation asymmetry were ‘co-asymmetric.' Quantitatively, the H2A.Z-positive immortal chromosome set showed elevated H3K27me3 and elevated H3K4me3 detection, respectively, compared with the opposing H2A.Z-negative chromosome set. In contrast, binucleated cells with configurations of symmetric H2A.Z:asymmetric H3 trimethylation (Figures 2a, b and d, quadrant 4) and asymmetric H2A.Z:symmetric H3 trimethylation (Figures 2a, b and d, quadrant 1) were infrequent; and anti-asymmetric binucleated cells (that is, with H2A.Z-positive immortal chromosomes, but elevated trimethylation detection on mortal chromosomes) were not observed.

By transitive logic, binucleated cells showing H2A.Z:H3K27me3 co-asymmetry and H2A.Z:H3K4me3 co-asymmetry must also be co-asymmetric for H3K27me3 *and* H3K4me3. On the basis of quantitative analyses presented in Figure 2, we estimate that essentially all cells undergoing nonrandom co-segregation are co-asymmetric for elevated H2A.Z and H3K37me3 detection on immortal chromosomes; and at least ~60% of these binucleated cells are also co-asymmetric for elevated H3K4me3 detection on immortal chromosomes as well.

**Figure 3:**
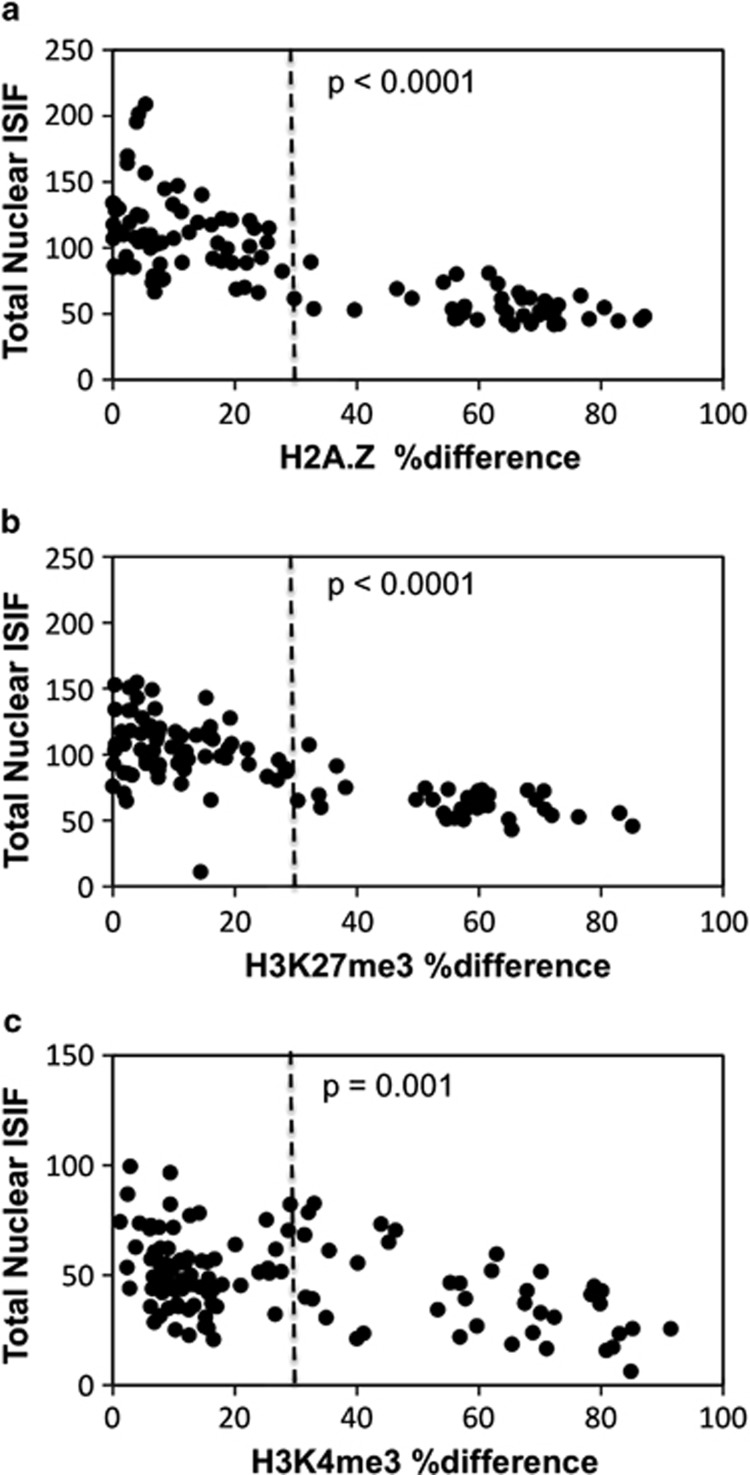
Murine hair follicle DSCs undergoing nonrandom co-segregation have a lower degree of H3K27me3 and H3K4me3 detection. The total nuclear ISIF of strain 3C5 binucleated cells, described in Figure 2 for conditions that promote nonrandom co-segregation (Xn-free), was evaluated with respect to their corresponding %difference in the mean fluorescence intensity of sister nuclei for (**a**), H2A.Z; (**b**), H3K27me3; and (**c**), H3K4me3. The vertical dotted line at 30% difference demarcates the separation between binucleated cells with a symmetric (left of line) *versus* an asymmetric (right of line) chromosomal detection pattern, as described in the legend of Figure 2. *P*-values indicate the level of statistical confidence, from the Student's unpaired *t*-test, that the observed lower mean total nuclear fluorescence of asymmetric binucleated cells (%difference*>*30), compared with symmetric binucleated cells (%difference⩽30), is not due to chance

**Figure 4:**
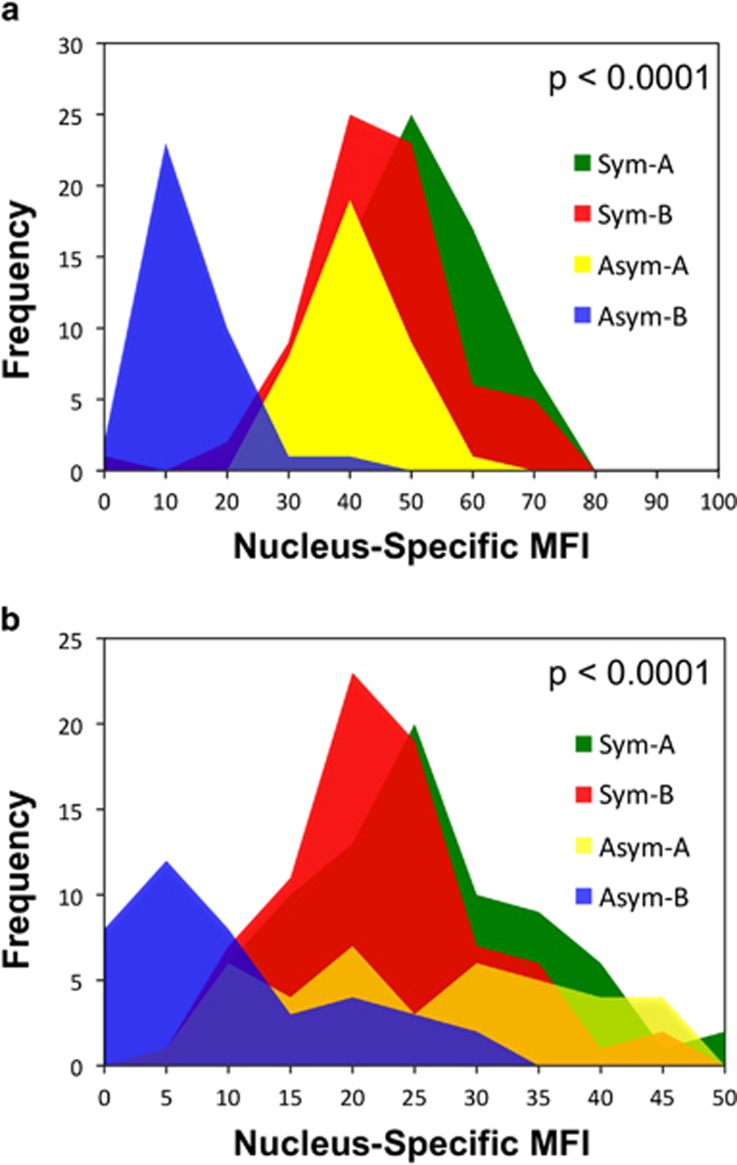
Mortal chromosomes in nonrandomly co-segregating murine hair follicle DSCs have a significantly reduced level of H3K27me3 and H3K4me3 detection. The mean fluorescence intensity (MFI) of four distinct groups of nuclei from the ISIF analyses of strain 3C5 binucleated cells, described in Figure 2 for conditions that promote nonrandom co-segregation (Xn-free), were used for these analyses. In each pair of nuclei in binucleated cells defined with either an asymmetric (Asym) or symmetric (Sym) chromosomal detection pattern, the nucleus with the greater MFI was designated as the ‘A' nucleus; and the nucleus with lesser MFI, as the ‘B' nucleus. The MFI distributions for each respective nuclear type (nuclear-specific MFI; Sym-A, Sym-B, Asym-A, Asym-B) are overlaid for comparison. (**a**) analysis for H3K27me3; (**b**) analysis of H3K4me3. *P*-values indicate the level of statistical confidence, from the Student's unpaired *t*-test, that the observed lower mean of the MFI distribution of Asym-B nuclei, compared with Sym-B nuclei, is not due to chance

To confirm that H3K27Kme3 asymmetry and H3K4me3 asymmetry were due to lower detection on mortal chromosomes compared with levels on randomly segregated chromosomes in symmetrically self-renewing cells, we evaluated the total MFI of binucleated cells with respect to their %difference in MFI (Figure 3). Similar to H2A.Z, which we reported earlier to have reduced detection on mortal chromosomes^6^ (Figure 3a), binucleated cells with H3K27me3 asymmetry and H3K4me3 asymmetry exhibited a significantly lower total MFI (Figures 3b and c, respectively). Respectively, the average MFI of binucleated cells showing asymmetric chromosomal detection decreased significantly by 49, 38, and 23% (Figures 3a–c, respectively;>30% difference) compared with binucleated cells showing symmetric chromosomal detection (Figure 3,⩽30% difference). Figure 4 data show that the reduced average level of detection is accounted for by the lower detection of H3K27me3 (Figure 4a) and H2K4me3 (Figure 4b) specifically on one set of chromosomes, which correspond to the mortal chromosome set (that is, H2A.Z-negative); whereas the immortal chromosomes (that is, H2A.Z-positive) retain a detection level comparable to randomly segregated chromosomes.

## Discussion

The observed global co-asymmetry of increased H3K27me3 and H3K4me3 detection on immortal chromosomes is reminiscent of the gene-specific bivalency of these epigenetic marks that have been reported for embryonic cells,^27^ embryonic stem cells,^8, 9, 27, 28^ and DSCs, including hematopoietic DSCs,^29^ neural DSCs,^9^ and primordial germ DSCs.^30^ In particular, this bivalent state is considered to hold in check transcription of genes involved in the developmental regulation and tissue-specific cell differentiation that are ‘poised' in stem cells for initiation of developmental programs that occur with conversion to monovalent states. The global reduction of H3K27me3 and H3K4me3 detection on mortal chromosomes in nonrandomly co-segregating mouse hair follicle DSCs may reflect a similar, except asymmetric, activation of previously bivalent genes that is restricted to the chromosomes that will segregate to lineage-committed sisters produced during asymmetric self-renewal by DSCs.

An important consideration for interpretations of all epigenomic studies using immunodetection is the possibility that failure to detect a given antigenic epitope could be due to either its physical absence or its molecular masking. Though usually unable to assess or overlooked, this qualification applies to all chromatin immunoprecipitation-based assays, as well as the findings presented here. In fact, previously, we have found that H2A.Z chromosomal asymmetry during nonrandom co-segregation is due to molecular masking.^6^ In the present studies, so far, we have not identified mildly denaturing conditions that preserve H3K27me3 and H3K4me3 antigenic epitopes on immortal chromosomes, so that we can look for masking of the trimethylations on mortal chromosomes. Therefore, a molecular masking mechanism cannot be excluded at this time. However, whether the epigenetic marks are reduced or masked, our findings reveal a significant new property of nonrandomly co-segregating chromosomes in DSCs that is likely to manifest mechanisms of asymmetric DSC fate specification and/or mechanisms of nonrandom co-segregation. In addition, the co-elevation of H3K27me3 and H3K4me3 on the chromosomes of asymmetrically self-renewing DSCs, compared with their lineage-committed sisters, may constitute a new specific biomarker for DSC identification in tissues.

An important issue that this first study cannot address is whether the findings apply to DSCs in tissues in general. There have been several reports of failure to detect nonrandom co-segregation *in vitro* cell populations enriched for specific stem types^31^ or in specific stem cell populations *in vivo*.^32, 33, 34, 35^ In the latter case, reported *in vivo* investigations of mouse skin hair follicles^32, 33^ were contradicted by the expansion of cells from mouse whisker follicles, which exhibit nonrandom co-segregation in cell culture.^7^ We have commented previously on possible technical explanations for these and other discrepancies^7, 36^ (ref. 35, online comment).

As a biological consideration, there is still quite limited knowledge of how mammalian DSC maintenance programs may differ among different tissues. On the basis of our *ex vivo* studies, we have proposed that deterministic asymmetric self-renewal and associated nonrandom co-segregation predominate *in vivo*.^37^ During deterministic asymmetric self-renewal, individual DSCs divide asymmetrically. However, this hypothesis is at odds with a growing body of work that is consistent with the view that neutral competition is the cellular basis for DSC asymmetric self-renewal in tissues.^38^ When it has been possible to define the asymmetric self-renewal pattern of DSCs undergoing nonrandom co-segregation, it has been deterministic.^7^ Asymmetric self-renewal by neutral competition does not require that individual DSCs divide asymmetrically. Theoretically, with neutral competition, nonrandom co-segregation would be ineffective for preserving DSC genetic fidelity.^37^ However, it could still be effective in determining DSC's fate stochastically, as is required for DSC compartment maintenance by neutral competition.^38^

Here, it is important to emphasize that experimental data consistent with neutral competition, though very well developed, cannot and does not exclude deterministic asymmetric self-renewal by DSCs.^38, 39^ So, the many examples of nonrandom co-segregation detected *in vivo*, *ex vivo,* and *in vitro* could occur in tissues maintained by neutral competition or deterministic asymmetric self-renewal. If neutral competition is responsible, the asymmetric reduction in the detection of H3K27me3 and H3K4me3 on mortal chromosomes could signify a function in stochastic DSC fate determination. If deterministic asymmetric self-renewal is responsible, the reduction could indicate important aspects of mechanisms for DSC fate determination and genetic fidelity. We favor the latter explanation as a universal feature of DSCs in the majority of postnatal mammalian tissues.^37^

## Materials and methods

Mouse hair follicle DSCs were maintained as previously described.^7^ Cells were fixed at room temperature with 3.7% paraformaldehyde/phosphate-buffered saline (PBS) and permeabilized with 0.2% Triton X-100/PBS for 10 min each, followed by blocking with 10% normal goat serum in PBS normal goat serum (NGS). Blocked cells were incubated with mouse anti-H3K27me3 monoclonal antibodies (Cat# ab6002, Abcam, Cambridge, MA, USA; diluted 1 : 100 in 10% NGS, 1 h, room temperature) or mouse anti-H3K4me3 monoclonal antibodies (Cat# ab12209, Abcam; diluted 1 : 200 in 10% NGS, 16–24 h, 4 °C). Thereafter, slides were washed with 0.5% bovine serum albumin (BSA) in PBS and incubated at room temperature for 1 h with Alexa Fluor 568 nm-conjugated secondary goat anti-mouse IgG antibodies (Cat# A-11004, Invitrogen, Inc., Carlsbad, CA, USA) diluted 1 : 500 in 10% NGS. Thereafter, slides were washed with 0.5% BSA and incubated for 16–24 h at 4 °C with rabbit anti-H2A.Z polyclonal antibodies (Cat# 2718, Cell Signaling Technology, Danvers, MA, USA) diluted 1 : 200 in 10% NGS. Slides were then washed with 0.5% BSA and incubated at room temperature for 1 h with Alexa Fluor 488-conjugated secondary goat anti-rabbit IgG antibodies (Cat# A-11008, Invitrogen, Inc.) diluted 1 : 300 in 10% NGS. Continuing, slides were washed with 0.5% BSA and mounted with DAPI-containing VectaShield mounting media. Peptide epitope antigens for specificity blocking experiments were purchased from Abcam (H3K27me3 peptide: Cat# ab1782; H3K4me3 peptide: Cat# ab1342). Epifluorescence images were captured with a Leica DMR microscope and Leica DC300F digital camera system. The mean pixel intensity of fluorescent nuclei was quantified using NIH Image J software.^6, 7^ The %difference in MFI between sister nuclei was calculated as ((A-B)/A) × 100%, where A⩾B. Total nuclear fluorescence equals A+B. The statistical significance of observed differences in categorical data was evaluated in 2 × 2 contingency tables using Fisher's two-tailed exact test. Student's *t*-test was used to evaluate the statistical significance of differences in the MFI of compared groups of nuclei.

## Abbreviations

Asym: asymmetric
BSA: bovine serum albumin
CD: cytochalasin D
ChIP: chromatin immunoprecipitation
DAPI: 4'-6-diamido-2-phenylindole
DSC: distributed stem cell
H3K27me3: trimethylated histone H3 lysine 27
H3K4me3: trimethylated histone H4 lysine 4
ISIF: *in situ* immunofluorescence
MFI: mean fluorescence intensity
NGS: normal goat serum
PBS: phosphate-buffered saline
SACK: suppression of asymmetric cell kinetics
Sym: symmetric
Xn: xanthine
