# Supplementary material for: Decreased H3K27 and H3K4 trimethylation on mortal chromosomes in distributed stem cells
Source: Cell Death Dis. 2014 Dec 4;5(12):e1554–. doi: 10.1038/cddis.2014.522 (PMC4649838; doi:10.1038/cddis.2014.522)
Supplement: Supplementary Information [file cddis2014522x1.pdf]

Supplementary Information

Antigen-blocking experiments were performed with respective peptide epitope antigens for anti-H3K27me3 and anti-H3K4me3 antibodies. For these studies, 3C5 hair follicle distributed stem cells were grown under optimal conditions for asymmetric self-renewal and arrested with cytochalasin D (CD). As shown in Fig. S1A, pre-incubation of the anti-H3K27me3 antibodies at room temperature for 1 hour with 0.1 microgram of H3K27me3 peptide epitope antigen (Cat# ab1782; Abcam, Cambridge, MA) ablated anti-H3K27me3-dependent fluorescence. Similarly, pre-incubation of the anti-H3K4me3 antibodies at room temperature for 1 hour with 0.1 microgram of H3K4me3 peptide epitope antigen (Cat# ab1342; Abcam, Cambridge, MA) ablated anti-H3K4me3-dependent fluorescence (Fig. S1B).

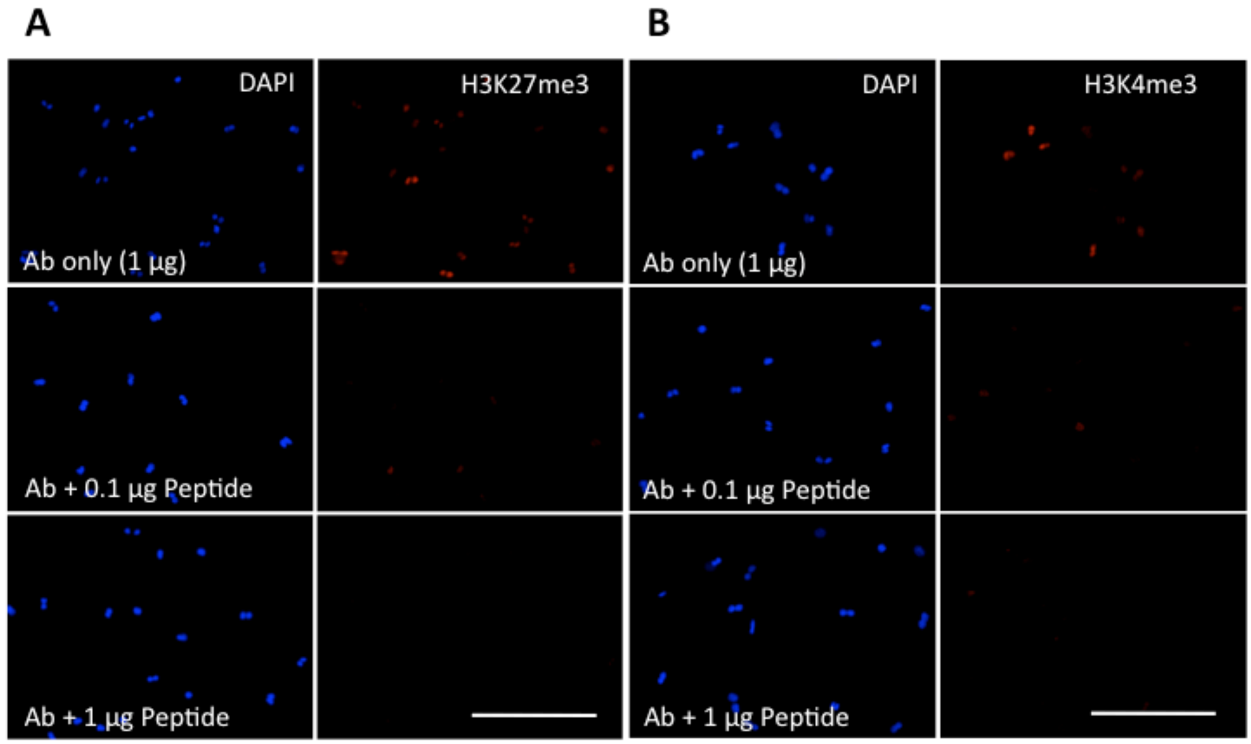

Figure S1. Peptide epitope antigen-blocking analyses to confirm the specificity of anti-H3K27me3 and anti-H3K4me3 antibodies in CD arrest studies. ISIF was performed against CD-arrested asymmetrically self-renewing 3C5 hair follicle distributed stem cells applying either anti-H3K27me3 (**A**) or anti-H3K4me3 antibodies (**B**). Detection of the respective nuclear antigens with the unblocked antibodies was compared to detection after antibodies were pre-blocked with 0.1 µg or 1.0 µg of the corresponding peptide epitope antigen. DAPI fluorescence identified cell nuclei. **Scale bar** = 200 µm.
